# Energy Taxis toward Host-Derived Nitrate Supports a *Salmonella* Pathogenicity Island 1-Independent Mechanism of Invasion

**DOI:** 10.1128/mBio.00960-16

**Published:** 2016-07-19

**Authors:** Fabian Rivera-Chávez, Christopher A. Lopez, Lillian F. Zhang, Lucía García-Pastor, Alfredo Chávez-Arroyo, Kristen L. Lokken, Renée M. Tsolis, Sebastian E. Winter, Andreas J. Bäumler

**Affiliations:** a Department of Medical Microbiology and Immunology, School of Medicine, University of California at Davis, Davis, California, USA; b Departamento de Genética, Universidad de Sevilla, Seville, Spain; c Department of Microbiology, University of Texas Southwestern Medical Center, Dallas, Texas, USA

## Abstract

*Salmonella enterica* serovar Typhimurium can cross the epithelial barrier using either the invasion-associated type III secretion system (T3SS-1) or a T3SS-1-independent mechanism that remains poorly characterized. Here we show that flagellum-mediated motility supported a T3SS-1-independent pathway for entering ileal Peyer’s patches in the mouse model. Flagellum-dependent invasion of Peyer’s patches required energy taxis toward nitrate, which was mediated by the methyl-accepting chemotaxis protein (MCP) Tsr. Generation of nitrate in the intestinal lumen required inducible nitric oxide synthase (iNOS), which was synthesized constitutively in the mucosa of the terminal ileum but not in the jejunum, duodenum, or cecum. Tsr-mediated invasion of ileal Peyer’s patches was abrogated in mice deficient for *Nos2*, the gene encoding iNOS. We conclude that Tsr-mediated energy taxis enables *S*. Typhimurium to migrate toward the intestinal epithelium by sensing host-derived nitrate, thereby contributing to invasion of Peyer’s patches.

## IMPORTANCE

Nontyphoidal *Salmonella* serovars, such as *S. enterica* serovar Typhimurium, are a common cause of gastroenteritis in immunocompetent individuals but can also cause bacteremia in immunocompromised individuals. While the invasion-associated type III secretion system (T3SS-1) is important for entry, *S*. Typhimurium strains lacking a functional T3SS-1 can still cross the intestinal epithelium and cause a disseminated lethal infection in mice. Here we observed that flagellum-mediated motility and chemotaxis contributed to a T3SS-1-independent pathway for invasion and systemic dissemination to the spleen. This pathway required the methyl-accepting chemotaxis protein (MCP) Tsr and energy taxis toward host-derived nitrate, which we found to be generated by inducible nitric oxide synthase (iNOS) in the ileal mucosa prior to infection. Collectively, our data suggest that *S*. Typhimurium enhances invasion by actively migrating toward the intestinal epithelium along a gradient of host-derived nitrate emanating from the mucosal surface of the ileum.

## INTRODUCTION

*Salmonella enterica* serovar Typhimurium causes a disseminated infection in genetically susceptible mice, which is commonly used to study the pathogenesis of typhoid fever (mouse typhoid model) (summarized in reference 1). *S*. Typhimurium initiates infection of mice by preferentially invading the intestinal epithelium of Peyer’s patches in the terminal ileum (2), followed by dissemination of the pathogen to internal organs, such as the spleen, where it replicates within macrophages (3).

Modeling of epithelial invasion in the 1980s using cultured epithelial cell lines identified flagellum-mediated motility (4) and the invasion-associated type III secretion system (T3SS-1) (5) encoded by *Salmonella* pathogenicity island 1 (SPI1) (6) as potential virulence factors contributing to epithelial entry. An initial characterization of these potential virulence factors in the mouse typhoid model suggested that inactivation of flagellum biosynthesis genes causes less attenuation (2- to 9-fold) (7) than inactivation of T3SS-1 biosynthesis genes (16- to 60-fold) (5, 8, 9). This early work helped erect the concept that T3SS-1 represents the main virulence factor for mucosal invasion, while flagella are not a major contributor to *Salmonella* pathogenesis. Consequently, subsequent work focused largely on elucidating the mechanism underlying T3SS-1-mediated epithelial entry*.*

However, recent studies suggest that flagella play a more significant role during the interaction of *S*. Typhimurium with its vertebrate host than previously appreciated. The development of models for *S*. Typhimurium-induced gastroenteritis revealed that flagellum-mediated motility contributes to the development of intestinal inflammation in calves (10) and in streptomycin-pretreated mice (mouse colitis model) (11). Furthermore, flagellum-mediated motility is required for driving a luminal expansion of *S*. Typhimurium during colitis (12, 13). These observations suggest that it might be warranted to reconsider the contribution of flagella to *Salmonella* pathogenesis.

There is also reason to believe that T3SS-1-mediated invasion is not the sole pathway for *S*. Typhimurium to cross the epithelial barrier in the ileum. That is, studies on the spread of *S*. Typhimurium in the mouse typhoid model identified a T3SS-1-independent pathway contributing to early dissemination of the pathogen from the intestinal lumen to the spleen (14). However, the *S*. Typhimurium virulence factors contributing to this T3SS-1-independent route of entry remain to be identified.

Here we investigated whether flagellum-mediated motility contributes to a T3SS-1-independent pathway of Peyer’s patch invasion and studied the underlying mechanism.

## RESULTS

### Flagella and T3SS-1 independently contribute to Peyer’s patch invasion and dissemination to the spleen.

To investigate the contribution of flagella and T3SS-1 to invasion and dissemination in the mouse typhoid model, groups of mice were infected intragastrically with an *S*. Typhimurium wild-type strain (IR715), a nonmotile mutant lacking both flagellin genes of *S*. Typhimurium (*fliC fljB* mutant [SW473]), a T3SS-1-deficient mutant (*invA* mutant [SW399]), and a strain lacking both flagella and T3SS-1 (*invA fliC fljB* mutant [FR90]). We reasoned that if flagella and T3SS-1 contribute to the same invasion pathway, the invasion defect observed for an *invA fliC fljB* mutant should be similar to that of an *invA* mutant, because the mutation in *invA* would epistatically mask the phenotype caused by mutations in *fliC* and *fljB*. Alternatively flagella and T3SS-1 might each contribute to a different invasion pathway, which would predict that combining the *invA*, *fliC*, and *fljB* mutations would yield a stronger phenotype than produced by either an *invA* mutant or an *fliC fljB* mutant.

**FIG 1:**
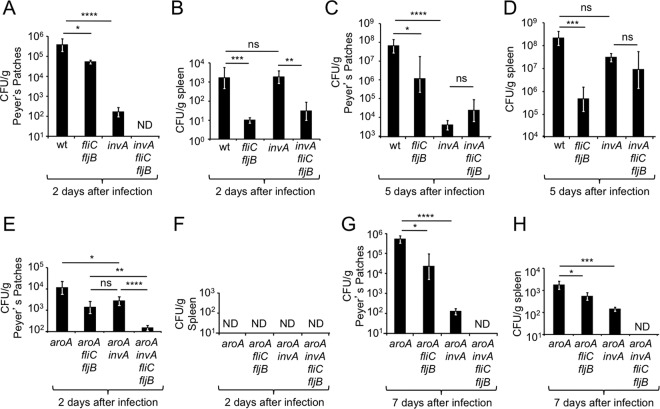
Flagella contribute to a T3SS-1-independent pathway of dissemination during *S*. Typhimurium infection of mice. Groups of mice were infected intragastrically with the indicated *S*. Typhimurium strains, and organs were collected at the indicated time points after infection. Bars represent geometric means ± standard errors of CFU recovered from Peyer’s patches (A, C, E, and G) or the spleen (B, D, F, and H). ND, none detected; wt, *S*. Typhimurium wild type; ns, not statistically significantly different; *, *P* < 0.05; **, *P* < 0.01; ***, *P* < 0.005; ****, *P* < 0.0005.

Two days after infection, the *invA* mutant and the *fliC fljB* mutant were both recovered in significantly (*P* < 0.0005 and *P* < 0.05, respectively) lower numbers from Peyer’s patches than the *S*. Typhimurium wild type, suggesting that both flagella and T3SS-1 contribute to tissue invasion, as previously suggested. Interestingly, the *invA fliC fljB* mutant was not recovered from Peyer’s patches 2 days after infection, indicating that flagella and T3SS-1 contributed to parallel pathways of invasion (Fig. 1A). However, unlike in Peyer’s patches, an *invA fliC fljB* mutant was recovered in similar numbers to the *fliC fljB* mutant from the spleen 2 days after infection, which supported the alternate model that flagella and T3SS-1 contribute to the same invasion pathway (Fig. 1B). Similarly, the *invA fliC fljB* mutant was recovered in similar numbers to the *invA* mutant from both the Peyer’s patches (Fig. 1C) and the spleen (Fig. 1D) at 5 days after infection.

We reasoned that these contradictory results could be due to the fact that in addition to invasion of Peyer’s patches, bacterial numbers recovered from organs might reflect differences between strains in their ability to grow in tissue, an effect that would become more pronounced at later time points after infection. To eliminate the factor of bacterial growth in tissue, we repeated the above-described experiment with bacterial strains carrying a mutation in *aroA*. An *aroA* mutant is able to resist clearance but cannot multiply in tissue (15), thus eliminating complicating effects resulting from bacterial replication in organs of infected animals. Two days after infection, an *aroA invA fliC fljB* mutant (FR95) was recovered in significantly lower numbers from Peyer’s patches than either the *aroA invA* mutant (FR92) (*P* < 0.0005) or the *aroA fliC fljB* mutant (FR94) (*P* < 0.01) (Fig. 1E). At this time point, no bacteria could be recovered from the spleen (Fig. 1F). By day 7 after infection, an *aroA invA fliC fljB* mutant was recovered in significantly lower numbers than either an *aroA invA* mutant or an *aroA fliC fljB* mutant from the Peyer’s patches (Fig. 1G), the mesenteric lymph node (see Fig. S1A in the supplemental material), and the spleen (Fig. 1H). Similar recovery of each strain from colon contents suggested that differences in bacterial numbers recovered from tissue were not due to altered growth of bacteria in the intestinal lumen (see Fig. S1B). In summary, when bacterial growth in tissue was prevented by a mutation in *aroA*, recovery of bacteria from the Peyer’s patches and the spleen consistently supported the idea that flagellum-mediated invasion was independent of invasion mediated by T3SS-1, which suggested that these two virulence factors acted in two parallel pathways of invasion and dissemination.

**FIG 2:**
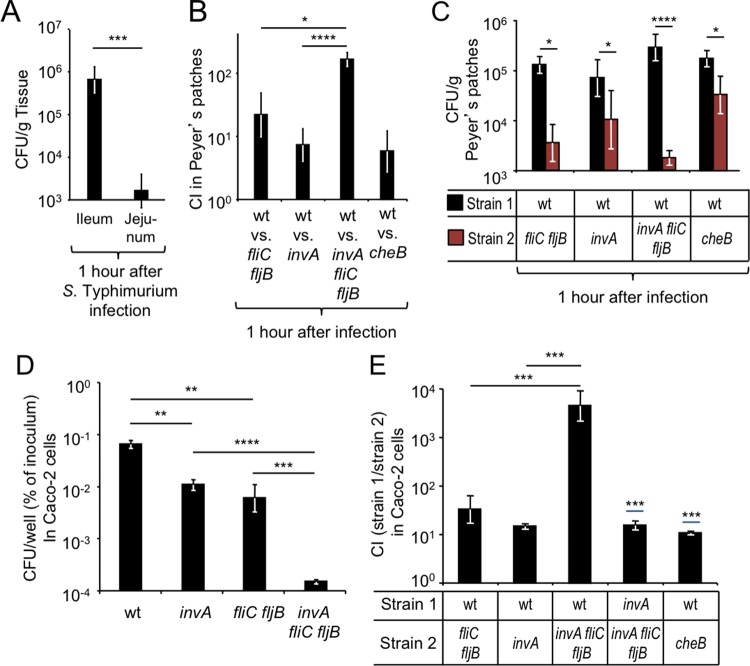
Flagella and chemotaxis contribute to epithelial invasion through a T3SS-1-independent pathway. (A to C) Groups of mice were infected intragastrically with the indicated *S*. Typhimurium strains or strain mixtures, and organs were collected at the indicated time points after infection. Bars represent geometric means ± standard errors of CFU (A and C) or the competitive index (CI) (B) recovered from Peyer’s patches. (D and E) The indicated *S*. Typhimurium strains (D) or strain mixtures (E) were allowed to invade model epithelia (polarized Caco-2 cells) for 30 min in an anaerobe chamber before extracellular bacteria were killed by treatment with gentamicin under aerobic conditions. Bars represent geometric means ± standard errors of gentamicin-resistant CFU recovered from model epithelia. *, *P* < 0.05; **, *P* < 0.01; ***, *P* < 0.005; ****, *P* < 0.0005; wt, *S*. Typhimurium wild type.

Next, we wanted to determine whether an early time point after infection would enable us to study invasion by virulent *S*. Typhimurium before growth in tissue could take place. A previous study suggested that bacteria could be recovered from Peyer’s patch tissue as early as 1 h after intragastric inoculation with *S. enterica* serovar Enteritidis (2). To determine whether invasion was detectable at this early time point, mice were infected with the *S*. Typhimurium wild type, and the ileum and jejunum were collected 1 h later and treated with gentamicin to kill extracellular bacteria residing in the intestinal lumen (gentamicin protection assay). Recovery of tissue-associated *S*. Typhimurium revealed marked invasion in the ileum but not in the jejunum (Fig. 2A), which was consistent with observations made with *S*. Enteritidis-infected mice (2).

To further investigate the contribution of *S*. Typhimurium virulence factors to invasion, mice were infected with a 1:1 mixture of the *S*. Typhimurium wild type and either an *fliC fljB* mutant, an *invA* mutant, or an *invA fliC fljB* mutant. One hour after infection, ileal Peyer’s patches were collected and incubated with gentamicin to kill extracellular bacteria. The wild type was recovered in higher numbers from ileal Peyer’s patches than either an *fliC fljB* mutant or an *invA* mutant (Fig. 2B and C), suggesting that both flagella and T3SS-1 contributed to invasion. However, the fitness advantage of the wild type over an *invA fliC fljB* mutant was significantly larger than that over an *fliC fljB* mutant (*P* < 0.05) or an *invA* mutant (*P* < 0.0005). These results provided further support for the hypothesis that flagella and T3SS-1 contributed to parallel pathways of ileal Peyer’s patch invasion.

We then wanted to investigate the mechanism by which flagella contributed to invasion of ileal Peyer’s patches at 1 h after infection. Flagellum-mediated motility is required for chemotaxis toward chemoattractants (reviewed in reference 16). We thus hypothesized that flagellum-mediated motility might be required by *S*. Typhimurium *in vivo* for locating the mucosal surface through chemotaxis. To test this idea, mice were infected with a 1:1 mixture of the *S*. Typhimurium wild type and a chemotaxis-deficient mutant (*cheB* mutant [AT349]), and bacteria were recovered from ileal Peyer’s patches 1 h after infection using a gentamicin protection assay. The *S*. Typhimurium wild type was recovered in approximately 8-fold-higher numbers than the *cheB* mutant from ileal Peyer’s patches (*P* < 0.05) (Fig. 2A and B), suggesting that chemotaxis was required for invasion.

We next sought to investigate whether the contribution of motility and chemotaxis to invasion could be observed in a tissue culture model of infection. Polarized human colonic epithelial (Caco-2) cells were transferred into an anaerobe chamber to mimic the limited oxygen availability characteristic of the intestinal lumen (≪7.6 mm Hg PO_2_ or ≪1% O_2_) (17), and bacterial invasion was allowed to proceed for 30 min before killing of extracellular bacteria with gentamicin. We recovered significantly higher numbers of bacteria from model epithelia infected with the *S*. Typhimurium wild type compared to model epithelia infected with either an *invA* mutant (*P* < 0.01) or an *fliC fljB* mutant (*P* < 0.01) (Fig. 2D). However, both an *invA* mutant (*P* < 0.0005) and a *fliC fljB* mutant (*P* < 0.005) were recovered in significantly higher numbers from Caco-2 cells than an *invA fliC fljB* mutant (Fig. 2D), suggesting that in this model, flagella and T3SS-1 also contributed to parallel pathways of invasion, which was similar to what was observed in mice.

Next, Caco-2 cells were infected with a 1:1 mixture of the *S*. Typhimurium wild type and either an *fliC fljB* mutant, an *invA* mutant, or an *invA fliC fljB* mutant. Similar to what we observed at early time points (i.e., 1 h) after infection of mice (Fig. 2B), recovery of intracellular bacteria from Caco-2 cells revealed that an *invA fliC fljB* mutant exhibited a significantly (*P* < 0.005) larger invasion defect than either an *fliC fljB* mutant or an *invA* mutant (Fig. 2E). Furthermore, an *invA* mutant was recovered in approximately 10-fold-higher numbers than an *invA fliC fljB* mutant when cells were infected with a 1:1 mixture of both strains (*P* < 0.005), showing that flagella contributed to invasion of Caco-2 cells independently of T3SS-1 (Fig. 2E). Finally, the *S*. Typhimurium wild type was recovered in approximately 10-fold-higher numbers than a chemotaxis-deficient *cheB* mutant when cells were infected with a 1:1 mixture of both strains (*P* < 0.005), suggesting that chemotaxis contributed to invasion in this model (Fig. 2E).

### Constitutive iNOS synthesis in the ileal mucosa is a source of luminal nitrate.

**FIG 3:**
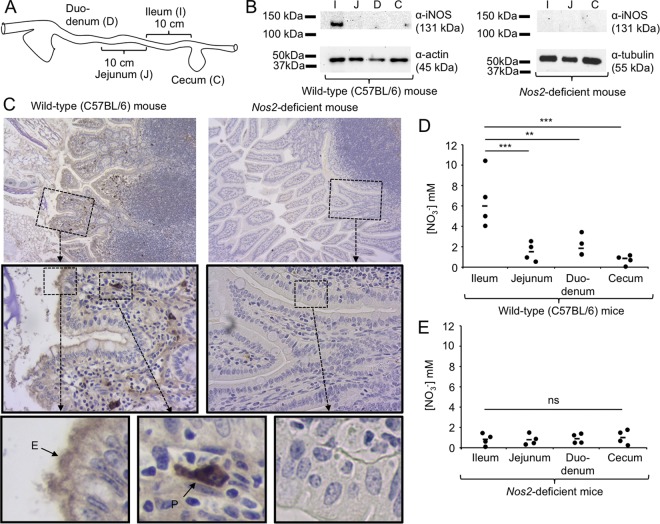
Constitutive iNOS synthesis is a source of host-derived nitrate in the murine ileum. (A) Image of the murine intestinal tract, indicating the origin of tissue samples analyzed in panel B. (B) Synthesis of iNOS in protein samples extracted from the indicated regions of the untreated murine gastrointestinal tract of wild-type (C57BL/6) mice or *Nos2*-deficient mice (*n* = 4) was detected by Western blotting using anti-mouse iNOS antibody (top panel). Detection of tubulin (right panel) or actin (left panel) by Western blotting served as a loading control. Each panel represents data from one representative animal. Molecular masses of standard proteins are indicated on the right. (C) Synthesis of iNOS in Peyer’s patches of the ileal mucosa from wild-type (C57BL/6) mice (left panels) or *Nos2*-deficient mice (right panels) was detected by immunohistochemistry (brown precipitate) in histological sections counterstained with hematoxylin (blue stain). Representative images captured with a 10× (top panels) or 40× (center panel) objective are shown. The bottom panel shows enlarged images of epithelial cells (E) and phagocytes (P). (D and E) The concentration of nitrate (NO_3_^−^) in luminal contents of the indicated regions of the gastrointestinal tract of wild-type (C57BL/6) mice (D) or *Nos2*-deficient mice (E) was detected was detected by a modified Griess assay. Bars represent geometric means, and each dot represents data from one animal. **, *P* < 0.01; ***, *P* < 0.005; ns, not statistically significantly different.

The results presented above sparked our interest in identifying possible signals that could direct chemotaxis of *S*. Typhimurium toward the mucosal surface of the ileum. A previous report suggests that constitutive inducible nitric oxide synthase (iNOS) synthesis is observed in the ileal mucosa of untreated mice but not in other parts of the murine gastrointestinal tract (18). We therefore investigated iNOS synthesis in different parts of the gastrointestinal tract of untreated mice using Western blotting. While iNOS synthesis was not detected in the duodenum, jejunum, or cecum of mice, we detected constitutive iNOS synthesis in the ileal mucosa (Fig. 3A and B). These data suggested that constitutive iNOS synthesis was a feature that differentiated the ileal mucosa from that in other parts of the murine gastrointestinal tract.

Next, we used immunohistochemistry to investigate the distribution and localization of iNOS synthesis in the ileal mucosa. No immunohistochemical signal was detected in mice deficient for *Nos2*, the gene encoding iNOS. In contrast, immunohistochemical detection of iNOS synthesis in wild-type (C57BL/6) mice generated a strong signal in phagocytes located in the lamina propria and a weaker signal at an apical location in epithelial cells lining the ileal mucosa (Fig. 3C).

We previously showed that nitric oxide (NO) generated by iNOS can react to form nitrate in the intestinal lumen (19, 20). To investigate whether constitutive iNOS synthesis in the ileal mucosa is a source of nitrate in the intestinal lumen, we measured nitrate concentrations in luminal contents from different parts of the gastrointestinal tract. Ileal contents contained on average 6 mM nitrate, which was significantly higher than the nitrate concentrations detected in contents from the jejunum (*P* < 0.005), duodenum (*P* < 0.01), or cecum (*P* < 0.005) of wild-type (C57BL/6) mice (Fig. 3D). In contrast, ileal contents from *Nos2*-deficient mice contained little nitrate (Fig. 3E), suggesting that elevated concentrations of nitrate in the ileal contents of wild-type mice were iNOS derived.

### Invasion of ileal Peyers’s patches requires Tsr-mediated chemotaxis toward nitrate.

**FIG 4:**
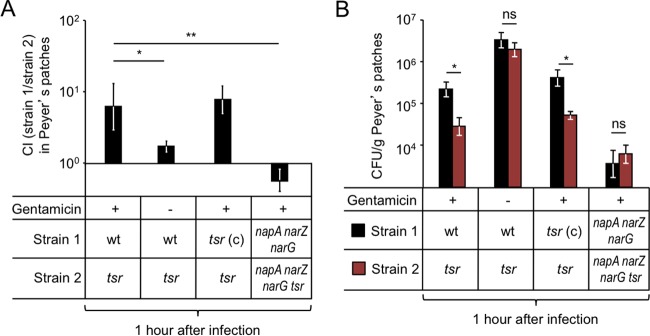
Energy taxis toward nitrate drives *S*. Typhimurium invasion of Peyer’s patches. Groups of mice were infected intragastrically with the indicated *S*. Typhimurium strain mixtures, and Peyer’s patches were collected at 1 h after infection. Bars represent geometric means ± standard errors of the competitive index (CI) (A) or CFU (B) recovered from Peyer’s patches. *, *P* < 0.05; **, *P* < 0.01; ns, not statistically significantly different; wt, *S*. Typhimurium wild type; *tsr* (c), *S*. Typhimurium *tsr* mutant (FR4) complemented with a plasmid (pFR6) carrying the intact *tsr* gene.

Respiratory electron acceptors, such as nitrate, serve as chemoattractants for *S*. Typhimurium (21, 22). The methyl-accepting chemotaxis protein (MCP) involved in sensing nitrate in the murine intestine is tsr (*t*axis to *s*erine and *r*epellents) (13). To investigate whether Tsr was required for invasion of the ileum, mice were infected with a 1:1 mixture of the *S*. Typhimurium wild type and a *tsr* mutant (FR4), and bacteria were recovered from ileal Peyer’s patches 1 h after infection using a gentamicin protection assay. The *S*. Typhimurium wild type was recovered in approximately 8-fold-higher numbers than the *tsr* mutant (*P* < 0.05) (Fig. 4A and B), suggesting that Tsr was required for invasion. When the experiment was repeated in the absence of gentamicin treatment, bacteria were recovered from Peyer’s patch homogenates at approximately 10-fold-higher numbers (Fig. 4B), implying that approximately 90% of bacteria were gentamicin sensitive (i.e., extracellular), presumably residing in the mucus layer associated with the luminal surface of Peyer’s patches. Importantly, in the absence of gentamicin treatment, the *S*. Typhimurium wild type was recovered in similar numbers to the *tsr* mutant (Fig. 4A), illustrating that Tsr did not confer a general fitness advantage in the lumen of the ileum. Finally, introduction of the cloned *tsr* gene on a plasmid (pFR6) into the *tsr* mutant (FR4) restored the defect in Peyer’s patch invasion 1 h after infection, as shown using a gentamicin protection assay (Fig. 4A and B). Collectively, these results support the idea that Tsr-mediated chemotaxis contributed to Peyer’s patch invasion.

Tsr mediates chemotaxis toward respiratory electron acceptors indirectly, by sensing the proton motive force, a process termed energy taxis (23–26). The *S*. Typhimurium genome encodes three nitrate reductases, encoded by the *napABC*, *narZYV*, and *narGHI* genes, which can use nitrate as a respiratory electron acceptor and generate proton motive force (19). Inactivation of *napA*, *narZ*, and *narG* abrogates nitrate reductase activity in *S*. Typhimurium, thereby preventing Tsr-mediated energy taxis toward nitrate (13). Thus, we wanted to determine whether inactivation of genes encoding nitrate reductases in *S*. Typhimurium could abrogate Tsr-mediated invasion of ileal Peyer’s patches. Mice were infected with a 1:1 mixture of a nitrate respiration-deficient mutant (*napA narZ narG* mutant [CAL50]) and a nitrate respiration-deficient mutant lacking Tsr (*napA narZ narG tsr* mutant [FR46]). Bacteria were recovered from ileal Peyer’s patches 1 h after infection using a gentamicin protection assay. Remarkably, in the absence of nitrate respiration, Tsr did not contribute to invasion of Peyer’s patches (Fig. 4A). Furthermore, inactivation of nitrate respiration caused a marked reduction in bacterial numbers recovered from Peyer’s patches (Fig. 4B). These data suggested that Tsr-mediated energy taxis toward nitrate contributed to invasion of ileal Peyer’s patches by *S*. Typhimurium.

### Tsr-mediated invasion of ileal Peyer’s patches is *Nos2* dependent.

**FIG 5:**
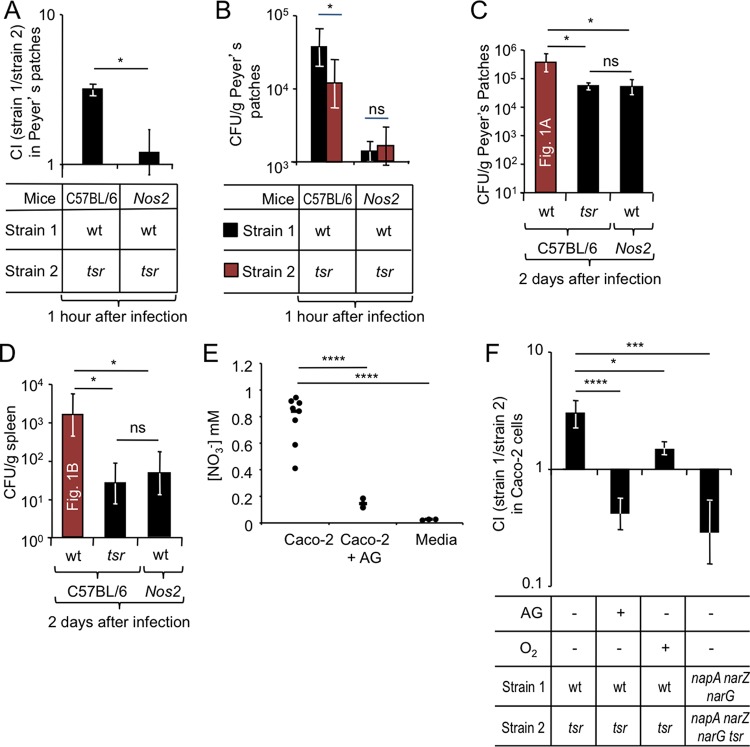
Host-derived nitrate drives a Tsr-dependent epithelial invasion of *S*. Typhimurium. (A to D) Groups of mice were infected intragastrically with the indicated *S*. Typhimurium strain mixtures (A and B) or strains (D and E), and organs were collected at the indicated time points after infection. Bars represent geometric means ± standard errors of the competitive index (CI) (A) or CFU (B to D) recovered from Peyer’s patches. (C and D) CFU of the *S*. Typhimurium wild type (wt) recovered from Peyer’s patches and spleen of C56BL/6 mice were determined in figure panels Fig. 1A and B, respectively, and are shown for comparison. (E) Nitrate concentration measured in the apical compartment of Transwell plates in the presence or absence of Caco-2 cells. (F) Model epithelia (polarized Caco-2 cells) were treated with the iNOS inhibitor aminoguanidine (AG) (+) or remained untreated (−) prior to *S*. Typhimurium infection. The indicated *S*. Typhimurium strain mixtures were allowed to invade model epithelia for 30 min in an anaerobe chamber (O_2_ −) or aerobically (O_2_ +) before extracellular bacteria were killed by treatment with gentamicin. Bars represent geometric means ± standard errors of gentamicin-resistant CFU recovered from model epithelia. *, *P* < 0.05; ***, *P* < 0.005; ****, *P* < 0.0005; ns, not statistically significantly different.

We next wanted to determine whether iNOS was required for Tsr-mediated invasion of ileal Peyer’s patches. To this end, wild-type (C57BL/6) mice and congenic *Nos2*-deficient mice were infected with a 1:1 mixture of the *S*. Typhimurium wild type and a *tsr* mutant, and bacteria were recovered from ileal Peyer’s patches 1 h after infection using a gentamicin protection assay. The *S*. Typhimurium wild type was recovered in higher numbers (*P* < 0.05) than the *tsr* mutant from Peyer’s patches of wild-type (C57BL/6) mice but not from Peyer’s patches of *Nos2-*deficient mice (Fig. 5A). Furthermore, *Nos2* deficiency caused a marked reduction in bacterial numbers recovered from Peyer’s patches (Fig. 5B).

To further characterize the role of iNOS in directing invasion, wild-type (C57BL/6) mice and *Nos2*-deficient mice were infected with a single *S*. Typhimurium strain, and bacteria were recovered from Peyer’s patches and the spleen 2 days later. A *tsr* mutant was recovered in significantly lower numbers (*P* < 0.05) from Peyer’s patches (Fig. 5C) and the spleen (Fig. 5D) of wild-type (C57BL/6) mice than from the *S*. Typhimurium wild type. Remarkably, recovery of the *S*. Typhimurium wild type from *Nos2*-deficient mice phenocopied numbers of the *tsr* mutant recovered from wild-type (C57BL/6) mice (Fig. 5C and D). Collectively, these data supported the idea that *Nos2* was required for Tsr-mediated invasion of Peyer’s patches.

### Tsr-mediated chemotaxis contributes to invasion of cultured model epithelia.

We next sought to investigate whether Tsr-mediated invasion could be observed in a tissue culture model of infection. Human intestinal epithelial Caco-2 cells synthesize low levels of iNOS constitutively, and iNOS synthesis further increases during infection (27). We thus wanted to determine whether a contribution to invasion of Tsr-mediated energy taxis toward nitrate could be detected using polarized Caco-2 cell monolayers (model epithelia) infected with *S*. Typhimurium in an anaerobe chamber as described above. Nitrate was detectable in the apical compartment of polarized Caco-2 cell cultures under these conditions (Fig. 5E). The *S*. Typhimurium wild type was recovered in significantly (*P* < 0.01) higher numbers than a *tsr* mutant when Caco-2 cells were infected with a 1:1 mixture of both strains (Fig. 5F). In the presence of the iNOS inhibitor aminoguanidine hydrochloride (AG), we detected significantly (*P* < 0.0005) lower nitrate levels from Caco-2 cell cultures compared to mock-treated Caco-2 cells (Fig. 5E), indicating that nitrate production was dependent on iNOS activity. Interestingly, a contribution of *tsr* to invasion was no longer observed when the invasion assay was performed in the presence of the iNOS inhibitor aminoguanidine hydrochloride (AG), suggesting that iNOS activity in Caco-2 cells was required for Tsr-dependent invasion.

Since inactivation of *napA*, *narZ*, and *narG* prevents Tsr-mediated energy taxis toward nitrate (13), we predicted that Tsr-mediated invasion would be abrogated by a genetic ablation of nitrate reductase activity in *S*. Typhimurium. Consistent with this idea, an *napA narZ narG* mutant was not recovered in higher numbers than an *napA narZ narG tsr* mutant when Caco-2 cells were infected with a 1:1 mixture of both strains (Fig. 5F). These data suggested that Tsr-mediated energy taxis toward nitrate contributed to invasion of Caco-2 cells in a low-oxygen environment.

In an aerobic environment, the proton motive force generated by oxygen respiration would be predicted to inhibit energy taxis toward nitrate (21, 23, 24). We thus reasoned that performing an invasion assay in a conventional 95% air (79% N_2_, 21% O_2_) atmosphere supplemented with 5% carbon dioxide (CO_2_) (19.95% O_2_ or 152 mm Hg PO_2_) would prevent Tsr-mediated invasion. Consistent with this prediction, a contribution of *tsr* to invasion was no longer observed after increasing oxygenation of the medium by performing the invasion assay outside an anaerobe chamber (Fig. 5F).

## DISCUSSION

Flagellum-mediated motility contributes markedly to *S*. Typhimurium invasion of epithelial cells *in vitro* (28–30). However, inactivation of flagellum biosynthesis genes causes little attenuation of *S*. Typhimurium in the mouse (7), while inactivation of T3SS-1 biosynthesis genes leads to a significant reduction in mouse virulence (5). These initial observations have focused subsequent research on studying T3SS-1-dependent invasion of epithelial cells by *S*. Typhimurium, which has provided numerous important molecular insights into the underlying mechanism (31–42). In contrast, the mechanism underlying flagellum-mediated invasion remains poorly characterized.

Based on *in vitro* studies, it has been proposed that flagellum-mediated motility merely increases the frequency of contact between pathogen and host cells to enhance T3SS-1-mediated invasion (43, 44). This model is not consistent with the observation that flagellum-mediated invasion was T3SS-1 independent, suggesting that it does not fully capture the sophisticated role motility plays during the interaction of *S*. Typhimurium with its host. Our results indicated that flagellum-mediated invasion of Peyer’s patches proceeded through a mechanism that was T3SS-1 independent. Previous work suggested that T3SS-1-independent mechanisms contribute to *S*. Typhimurium invasion in the mouse model (14); however, our report is the first to identify an *S*. Typhimurium virulence factor that participated in a T3SS-1-independent route of entry into Peyer’s patches. Flagellum-mediated invasion can be observed using cultured epithelial cells (4, 28–30), pointing to this cell type as a potential target for Tsr-mediated invasion *in vivo.*

Flagella enabled *S*. Typhimurium to migrate toward nitrate using energy taxis both *in vivo* and *in vitro*, but the latter required assay conditions that mimicked the limited oxygen availability in the gut. We found that nitrate needed for Tsr-mediated energy taxis was generated by iNOS synthesized by the host, suggesting that this mechanism likely enhanced invasion by enabling the pathogen to locate the mucosal surface *in vivo*. Dendritic cells can shuttle *S*. Typhimurium across the mucosal surface in a process that is T3SS-1 independent (14, 45), and it is possible that iNOS expressed in these phagocytes attracts the pathogen by energy taxis. Thus, additional work is needed to determine whether epithelial cells, phagocytes, or both cell types contribute to the flagellum-dependent, T3SS-1-independent pathway of Peyer’s patch invasion.

Energy taxis toward nitrate might contribute to a preference of *S*. Typhimurium for invading the ileal mucosa, because iNOS is expressed constitutively at this site, but not in other parts of the gastrointestinal tract (18). However, there are likely additional mechanisms contributing to the reported preferential invasion of ileal Peyer’s patches (2), such as a predilection of *S*. Typhimurium for invading M cells located in the follicle-associated epithelium of Peyer’s patches (46), the endocytic nature of M cells (47), and a thinner mucus layer overlaying Peyer’s patches (48, 49). Furthermore, there is a suppression of T3SS-1 synthesis in the cecum by a gradient of bile salts (50) and microbiota-derived propionate and butyrate (51). Nonetheless, our data suggest that energy taxis contributes to invasion of epithelial cells both *in vivo* and *in vitro* through a mechanism that is T3SS-1 independent, which illustrates that flagella play an important role in the pathogenic lifestyle of *S*. Typhimurium.

## MATERIALS AND METHODS

### Bacterial strains.

The plasmids and bacterial strains used in this study are listed in Tables 1 and 2, respectively. Unless indicated otherwise, bacteria were routinely cultured aerobically at 37°C in LB broth or on LB agar plates (BD Biosciences). Antibiotics were added to the media at the following concentrations: 0.03 mg/ml chloramphenicol, 0.1 mg/ml carbenicillin, 0.05 mg/ml kanamycin, 0.05 mg/ml nalidixic acid, and 0.01 mg/ml tetracycline (Tet).

**TABLE 1:** Bacterial strains used in this study

Strain	Genotype^a^	Source or reference
*S*. Typhimurium		
IR715	Nalidixic acid-resistant derivative of ATCC 14028	54
SL1346	*aroA554*::Tn*10*	55
SPN452	IR715 Δ*invA*::*tetRA* Δ*spiB*::KSAC (Tet^r^ Kan^r^)	56
SW399	IR715 *invA*::pSW127 (Carb^r^)	30
SW473	IR715 Δ*fliC*(−25 to +1494) *fljB5001*::MudJ	30
AT349	*cheB*::Tn*10* (Tet^r^)	57
AJB131	IR715 *aroA*::Tn*10* (Tet^r^)	This study
CAL50	IR715 Δ*napA* Δ*narZ narG*::pCAL5	19
FR4	IR715 *tsr*::pFR3 (Cm^r^)	13
FR46	IR715 *tsr*::pFR3 (Cm^r^) Δ*napA* Δ*narZ narG*::pCAL5	13
FR90	IR715 Δ*fliC*(−25 to +1494) *fljB5001*::MudJ *invA*::pSW127	This study
FR91	IR715 *aroA*::Tn*10* (Tet^r^) *TSR*::pFR3 (Cm^r^)	This study
FR92	IR715 *aroA*::Tn*10* (Tet^r^) *invA*::pSW127 (Carb^r^)	This study
FR93	IR715 *aroA*::Tn*10* (Tet^r^) *TSR*::pFR3 (Cm^r^) *invA*::pSW127 (Carb^r^)	This study
FR94	IR715 *aroA*::Tn*10* (Tet^r^) Δ*fliC*(−25 to +1494) *fljB5001*::MudJ	This study
FR95	IR715 *aroA*::Tn*10* (Tet^r^) Δ*fliC*(−25 to +1494) *fljB5001*::MudJ *invA*::pSW127	This study
*E. coli*		
DH5α λ*pir*	F^−^ *endA1 hsdR17* (r^−^ m^+^) *supE44 thi-1 recA1 gyrA96 relA1* Δ(*lacZYA*-*argF*)U169 *deoR nupG* ϕ80*lacZ*ΔM15 λ*pir*	58
S17-1 λ*pir*	*zxx*::RP4-2-Tet^r^::Mu-Kan^r^::Tn*7 recA1 thi pro hsdR* (r^−^ m^+^) λ*pir*	59
TOP10	F^−^ *mcrA* Δ(*mrr*-*hsdRMS*-*mcrBC*) φ80*lacZ*ΔM15 *lacX74 recA1 araD139* Δ(*ara-leu*)*7697 galE15 galK rpsL endA1 nupG*	Invitrogen

a Cm^r^, chloramphenicol resistance; Carb^r^, carbenicillin resistance; Kan^r^, kanamycin resistance.

**TABLE 2:** Plasmids used in this study

Designation	Relevant characteristic(s)	Source or reference
pCR2.1	Cloning vector	Invitrogen
pGP704	*ori*(R6K) *mobRP4* (Carb^r^)	60
pEP185.2	*ori*(R6K) *mobRP4* (Cm^r^)	61
pFR6	*tsr* open reading frame and promoter cloned into pWSK29	13
pSW127	′*invA*′ gene fragment cloned into pGP704, insertion of pSW127 into IR715 chromosome *invA* gene	30

### Generalized phage transduction.

Phage P22 HT *int-105* was used for generalized transduction. Transductants were routinely purified from phage contamination on Evans blue-uranine agar and then cross-streaked against P22 H5 to confirm phage sensitivity.

Using phage transduction, the *aroA554*::Tn*10* mutation from *S*. Typhimurium strain SL1346 was introduced into *S*. Typhimurium strains IR715 and SW473, generating strains AJB131 and FR94, respectively. The *invA*::pSW127 mutation from *S*. Typhimurium strain SW399 was introduced into *S*. Typhimurium strains SW473, AJB131, FR91, and FR94 to yield strains FR90, FR92, FR93, and FR95, respectively.

### Animal experiments.

All experiments in this study were approved by the Institutional Animal Care and Use Committee at the University of California at Davis.

### (i) Mouse typhoid model.

Female C57BL/6J mice or *Nos2*-deficient mice (B6.129P2-Nos2tm1Lau/J), aged 8 to 12 weeks, were obtained from The Jackson Laboratory (Bar Harbor, ME). For infections with individual *S*. Typhimurium strains or strain mixtures, groups (*n* = 4) of mice were inoculated with approximately 1 × 10^9^ CFU per animal. Animals were euthanized at the indicated time points to collect organs.

### (ii) One-hour infections.

For 1-h infections, groups (*n* = 4) of female C57BL/6J mice (Taconic Farms, Hudson, NY, or The Jackson Laboratory) or *Nos2*-deficient mice were inoculated with 0.1 ml of a suspension containing a 1:1 mixture containing 5 × 10^8^ CFU of each bacterial strain in LB broth. Approximately 1 h after infection, mice were euthanized, and a minimum of three Peyer’s patches were collected and incubated for 90 min in phosphate-buffered saline (PBS) containing gentamicin (100 µM). The ratio of both strains recovered from tissue (output ratio) was divided by the ratio present in the inoculum (input ratio) to determine the competitive index (CI).

### Caco-2 invasion assays.

Caco-2 cells were maintained on minimum essential medium (MEM) containing 10% fetal bovine serum (FBS). To polarize Caco-2 cells, 0.5 ml of medium containing approximately 1 × 10^5^ cells was seeded apically in 0.4-µm 12-mm Transwell plates (polycarbonate membrane [Corning-Costar]), and 1.0 ml of medium was added to the basolateral compartment. The medium was changed every 2 days, and the transepithelial electrical resistance was measured after a week and the day before the experiment using the Millicell-ERS electrical resistance system. Invasion assays were performed 2 weeks after seeding of Transwell plates with Caco-2 cells. The day before the experiment, bacterial cells were grown overnight under aerobic conditions and resuspended in PBS at a density of 10^9^ CFU/ml. Caco-2 cells were washed 2 times with PBS, 10 µl of bacterial cell culture was added to the apical compartment of Transwell plates in an anaerobe chamber, and the mixture was incubated for 30 min at 37°C. For aminoguanadine (AG) treatment, cells were incubated in PBS containing 1 mg/ml AG for 1 h before transfer into the anaerobe chamber and addition of bacterial cells. After 30 min of incubation, Transwell plates were removed from the anaerobe chamber, and cells were incubated in medium containing gentamicin (100 µM) for 90 min. Cells were washed three times with PBS, and intracellular bacteria were enumerated by spreading serial 10-fold dilutions of Caco-2 cell lysates (1% Triton X-100) on LB plates containing the appropriate antibiotics to determine CFU. Each assay was repeated at least three times independently.

### Nitrate measurements.

Intestinal nitrate measurements were performed as described previously with some modifications (52). Briefly, uninfected mice were euthanized, and the intestine was removed and divided along its sagittal plane. The mucus layer was gently scraped from the tissue and homogenized in 200 µl PBS and then placed on ice. Samples were centrifuged at 5,000 × *g* for 10 min at 4°C to remove the remaining solid particles. The supernatant was then filter sterilized (0.2-µm Acrodisc syringe filter [Pall Life Sciences, Port Washington, NY]). Measurement of intestinal nitrate followed an adaptation of the Griess assay. In this assay, nitrate was first reduced to nitrite by combining 50 µl of each sample with 50 µl of Griess reagent 1 containing vanadium(III) chloride (0.5 M HCl, 0.2 mM VCl_3_, 1% sulfanilamide) (53), and then the mixture was incubated at room temperature for 10 min. Next, 50 µl of Griess reagent 2 [0.1% (1-naphthyl)ethylenediamine dichloride] was added to each sample. Absorbance at 540 nm was measured immediately after the addition of Griess reagent 2 to detect any nitrite present in the samples. The samples were then incubated for 8 h at room temperature (to allow for reduction of nitrate to nitrite), and the absorbance at 540 nm was measured again. The initial absorbance was subtracted from the absorbance after 8 h to determine nitrate concentrations in the cecal mucus layer. Samples were tested in duplicate, and all measurements were standardized to the initial sample weight.

To measure nitrate in tissue culture assays, the day before experiment, polarized Caco-2 cell medium was changed to MEM containing 10% fetal bovine serum (FBS) medium without phenol. Supernatant was collected, and nitrate was measured using the Griess assay as described above.

### Western blotting.

The concentration of mouse protein from intestinal tissue was measured using a Pierce Micro bicinchoninic acid (BCA) protein assay kit in accordance with the manufacturer’s instructions. For each lane, a 0.02-mg sample of protein was boiled for 3 min and resolved by SDS-PAGE and then transferred from gels onto a polyvinylidene fluoride membrane (Millipore) by using a semidry transfer method (Bio-Rad Laboratories). Nonfat dried milk (2.5%) and Tween 20 (0.1% [Bio-Rad]) in a PBS solution were used as blocking agents. To detect iNOS, a 1:1,500 dilution of the primary antibody (mouse anti-mouse iNOS [BD Transduction Laboratories]) in blocking buffer was added to the membrane. As a loading control, tubulin or actin was detected with a 1:5,000 dilution of the primary antibody (rabbit anti-mouse α/β-tubulin and rabbit anti-mouse pan-actin [Cell Signaling]) in blocking buffer. A horseradish peroxidase-conjugated goat anti-mouse or goat anti-rabbit antibody (Bio-Rad) diluted 1:5,000 in blocking buffer was used as the secondary antibody. Chemiluminescence (Western Lightning Plus ECL chemiluminescent substrate [PerkinElmer]) was visualized by using a BioSpectrum (UVP) or G:Box (Syngene) imaging system. Raw images were processed with Photoshop CS2 (Adobe Systems) to uniformly adjust the brightness levels of the entire image.

### Immunohistochemistry.

To detect iNOS, a 1:1,500 dilution of the primary antibody (mouse anti-mouse iNOS [BD Transduction Laboratories]) in blocking buffer (2.5% dry milk, 0.1% Tween 20) was added to the tissue, and the mixture was incubated in a humidified chamber overnight at 4°C. Slides were then washed in phosphate-buffered saline (PBS) and then incubated with biotinylated secondary antibody for 20 min at room temperature, washed in PBS, and incubated with streptavidin-peroxidase complex (LSAB1 kit [Dako, Carpinteria, CA]) for 20 min at room temperature. The reaction was developed with a 0.024% diaminobenzidine solution (Dako) and counterstained with Mayer’s hematoxylin for 60 s.

### Statistical analysis.

Fold changes of ratios (bacterial numbers or mRNA levels) were transformed logarithmically prior to statistical analysis. An unpaired Student’s *t* test was used to determine whether differences in fold changes between groups were statistically significant (*P* < 0.05). A paired Student’s *t* test was used to determine whether differences in the recovery of two bacterial strains from the same animal (competitive infection) were statistically significant.

## SUPPLEMENTAL MATERIAL

Figure S1  Flagella contribute to a T3SS-1-independent pathway of dissemination. Groups of mice were infected intragastrically with the indicated *S*. Typhimurium strains, and organs were collected at the indicated time points after infection. Bars represent geometric means ± standard errors of CFU recovered from the mesenteric lymph nodes (MLN) (A) or the colon contents (B). ND, none detected; *, *P* < 0.05; ****, *P* < 0.0005. Download Figure S1, PDF file, 0.03 MB
