# Supplementary figures and images for: Energy Taxis toward Host-Derived Nitrate Supports a Salmonella Pathogenicity Island 1-Independent Mechanism of Invasion
Source: mBio. 2016 Jul 19;7(4):e00960-16. doi: 10.1128/mBio.00960-16 (PMC4958259; doi:10.1128/mBio.00960-16)

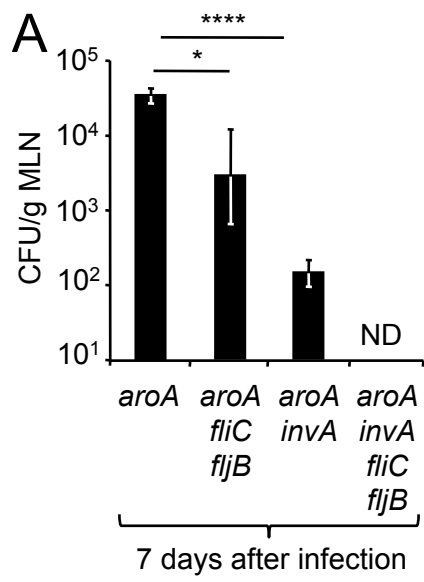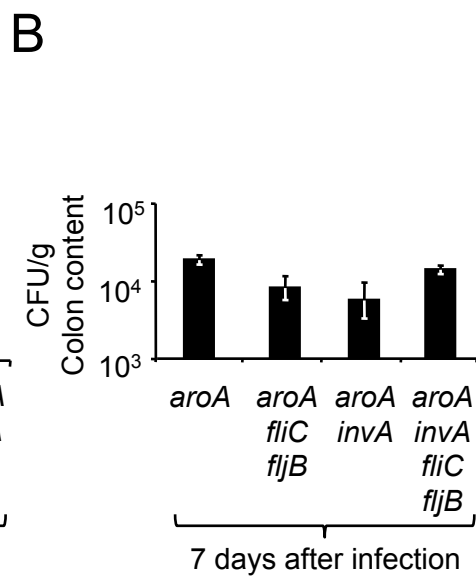

Supplement: Figure S1 — Flagella contribute to a T3SS-1-independent pathway of dissemination. Groups of mice were infected intragastrically with the indicated S. Typhimurium strains, and organs were collected at the indicated time points after infection. Bars represent geometric means ± standard errors of CFU recovered from the mesenteric lymph nodes (MLN) (A) or the colon contents (B). ND, none detected; *, P < 0.05; ****, P < 0.0005. Download [file mbo004162907sf1.pdf]
